# Supplementary material for: Obatoclax Rescues FUS-ALS Phenotypes in iPSC-Derived Neurons by Inducing Autophagy
Source: Cells. 2023 Sep 11;12(18):2247. doi: 10.3390/cells12182247 (PMC10527391; doi:10.3390/cells12182247)
Supplement: Supplementary file 1 [file cells-12-02247-s001.zip › 2023-09-07 supplemental information.pdf]

Supplemental Material

1. Supplementary Figures

- **Supplementary Figure S1.** Electrode-cell arrangements. Related to Figure 1.
- **Supplementary Figure S2.** Representative voltage traces and spike waveforms. Related to Figure 1.
- **Supplementary Figure S3.** Descriptive statistics of electrophysiological activity across time and pharmacological manipulation. Related to Figure 1.
- **Supplementary Figure S4.** Cell viability of the BH3 mimetics compounds. Related to Figure 1.
- **Supplementary Figure S5.** 1 nM obatoclax ameliorates FUS-eGFP SG formation. Related to Figure 1.
- **Supplementary Figure S6.** Effects of obatoclax on senescent cells. Related to Figure 2.
- **Supplementary Figure S7.** Stability of Obatoclax. Related to Figure 4.
- **Supplementary Figure S8.** Obatoclax induces autophagic efflux in P525L FUS-eGFP iPSC-derived neurons. Related to Figure 4.

2. Supplementary Tables

- **Supplementary Table S1.** Proteomics. Related to Figure 7.
- **Supplementary Table S2.** KEGG pathway analysis. Related to Figure 7.

3. Supplementary Experimental procedures

4. References

Supplementary Figures

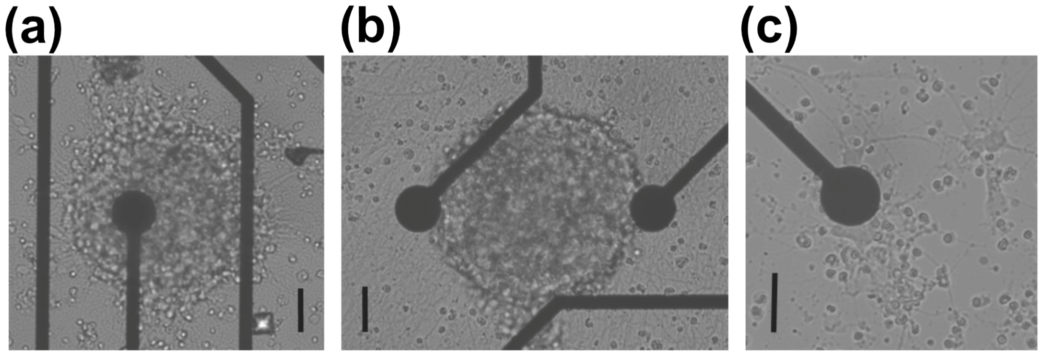

Supplementary Figure S1. Electrode-cell arrangements. Survival of cells was limited and live cell density was sparse when cultured on the multi-electrode array. **(a)** When cell aggregates formed directly above electrodes (n=3) electrophysiological activity was recorded in 100% of such arrangements and was stable throughout recording dates and drug conditions. **(b)** Other cell aggregates bordered electrodes (n=4). 25% of these configurations (n=1) yielded electrophysiological activity which was present on DIV21, but not DIV23. **(c)** When individual cells were located in direct proximity of an electrode (n=15), electrophysiological activity was recorded from one such configuration on DIV 23 only but then followed through the pharmacological manipulations. Electrophysiological data from this unit is highlighted with an open double arrow in Figure S3. Scale bars represent 40 μm. Related to Fig. 1.

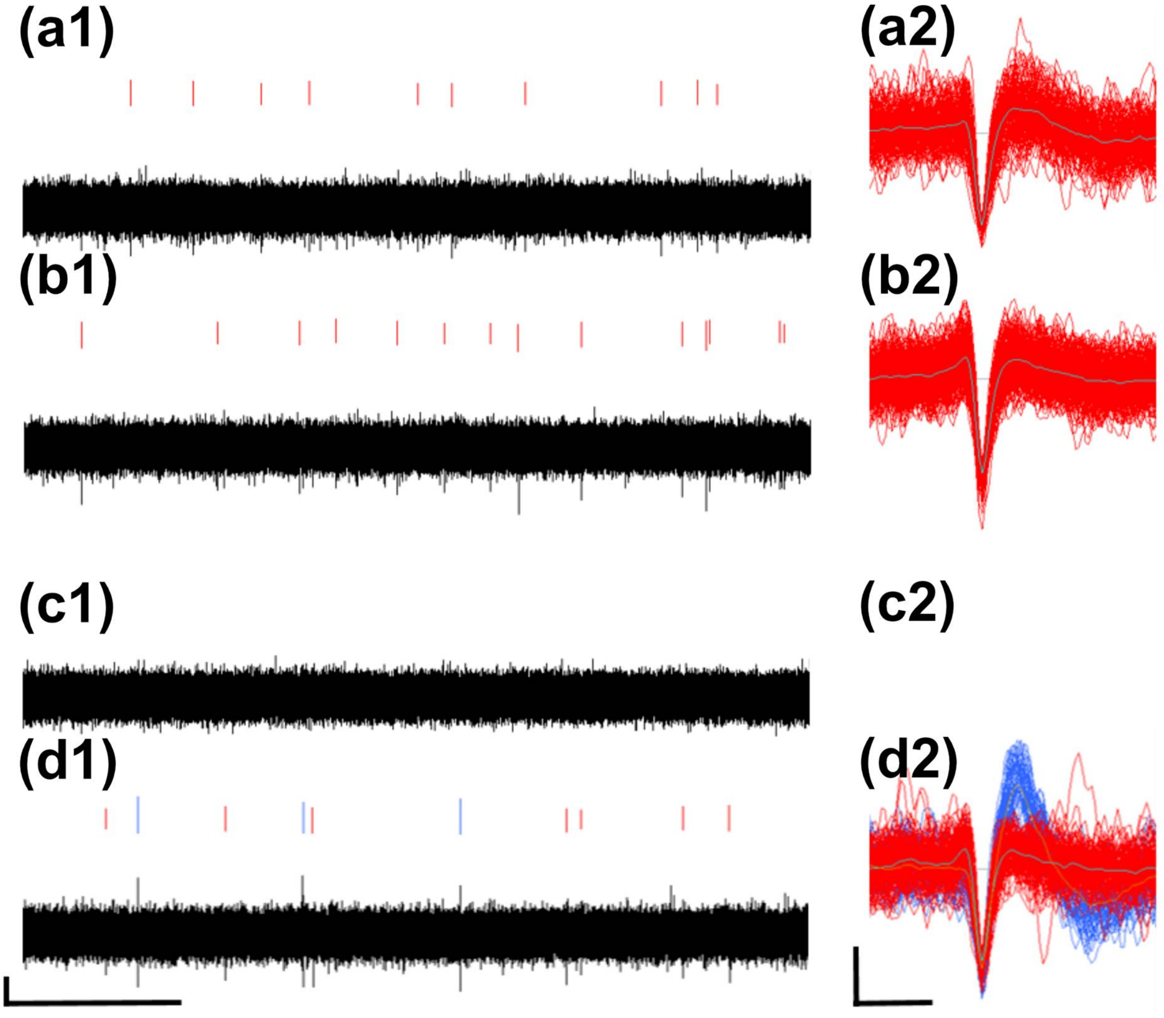

Supplementary Figure S2. Representative voltage traces and spike waveforms. Traces are taken from the sample highlighted with the filled arrow in Fig. 3. **(a)** DIV21. **(b)** DIV23, baseline. Action potential firing rates remained consistently below 1 Hz, but did not fall below 0.05 Hz. Mostly, non-differentiable multiunit activity was recorded. **(c)** DIV23 + tetrodotoxin (TTX). In the presence of TTX, no spiking activity was captured. **(d)** DIV23, wash out. In two recordings on DIV23, once during baseline and in a different electrode during wash out, distinguishable units were captured. However, they were conflated to multiunit activity of the respective electrode for the purpose of firing rate assessment. **(1)** Shows continuous voltage traces (bottom) with accompanied action potentials (top). Scale bars represent 10 μV and 5 s. **(2)** Shows peak aligned action potential waveforms. Scale bars represent 10 μV and 1 ms. Related to Fig. 1.

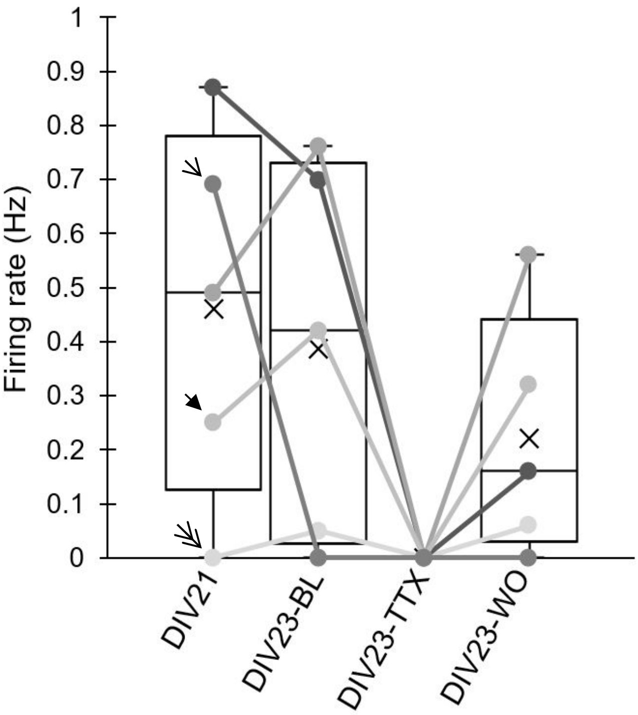

Supplementary Figure S3. Descriptive statistics of electrophysiological activity across time and pharmacological manipulation. Box plots conflate the five samples, from left to right, on DIV21, during baseline (BL), TTX and wash out on DIV23. Connected points in shades of gray represent individual data points over time. **(Open arrow)** Recordings from the left electrode in Fig. 1B. **Filled arrow)** Electrode-cell arrangement shown in Fig. 1A. Recordings shown in Fig. 2. **(Open double arrow)** Electrode-cell arrangement shown in Fig. S1C. Mean firing rates during baseline on DIV21 (0.46 ± 0.31 Hz) and DIV23 (0.39 ± 0.32 Hz) were comparable. The drop in mean firing frequency from DIV21 to DIV23 was mediated by a unit (open arrow) that was only captured firing intensely on DIV21, but was no longer present on DIV23. This could not be compensated by an incoming unit (open double arrow) due to its low firing rate. When the open arrowed unit is excluded from analysis, the mean firing rates on DIV21 and DIV23 are 0.40 ± 0.32 Hz and 0.48 ± 0.28 Hz, respectively. In the presence of TTX, no action potential firing could be detected. Following wash out of TTX, the mean firing rate returned, but remained attenuated (0.22 ± 0.20 Hz). Related to Fig. 1.

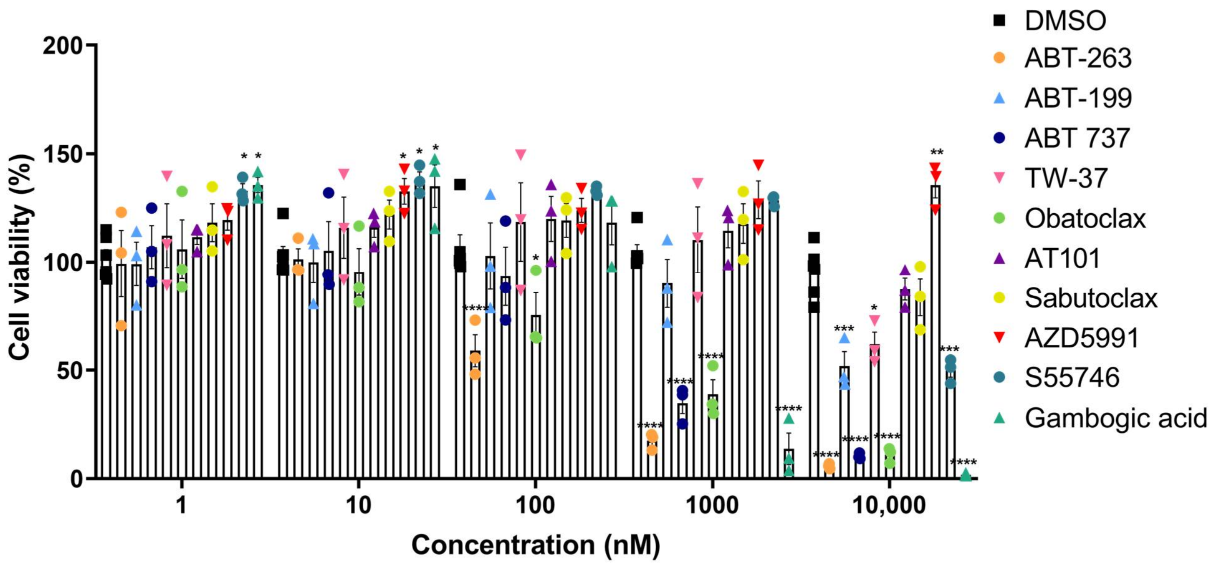

Supplementary Figure S4. Cell viability of the BH3 mimetics compounds. Compounds were tested at different concentrations for 24 hours. Cell viability was assessed with calcein AM-Red. The mean of three independent experiments (n=3), error bars indicate standard error of the mean (SEM). Treatments were analyzed and compared with treated with DMSO via two-way ANOVA with a Dunnet post-test, *, **, and **** indicate p <0.5, <0.01 and <0.0001, respectively. Related to Fig. 1.

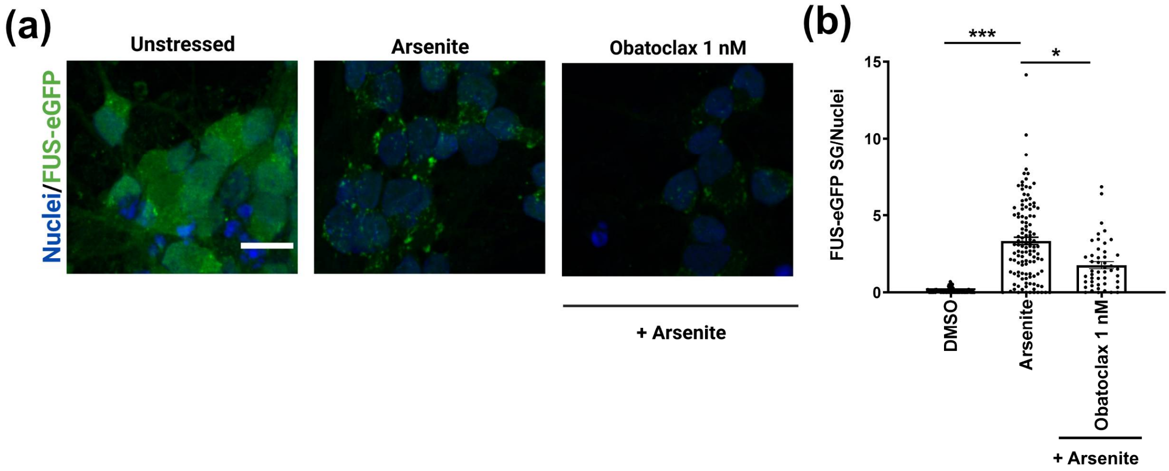

Supplementary Figure S5. 1 nM obatoclax ameliorates FUS-eGFP SG formation. Related to Figure 1. (a) Fluorescent confocal micrographs showing FUS-eGFP-positive SGs in different conditions in iPSC derived neurons. Scale bar = 10 μm. (b) The mean of three independent experiments (n=3), error bars indicate SEM. Treatments were analyzed via Kruskal-Wallis with a Dunn post-test. *, and *** indicate p <0.05 and <0.0001, respectively. Related to Fig. 1.

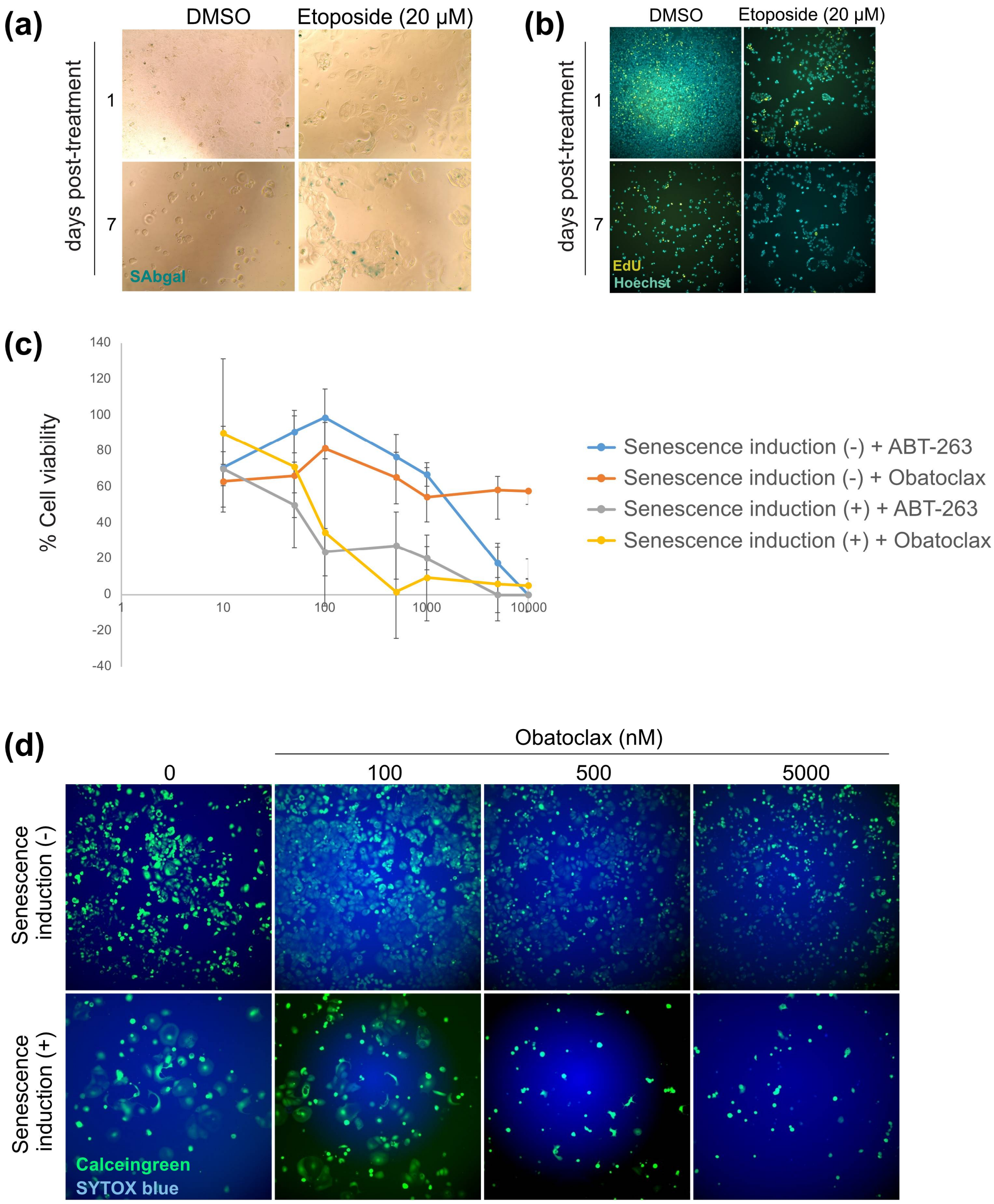

Supplementary Figure S6. Effects of obatoclax on senescent cells. (a) Etoposide-mediated induction of cell senescence in MCF-7 cells. Representative images of MCF-7 cells following senescence-associated β-galactosidase staining at the indicated times after treatment with 20 μM etoposide or vehicle (DMSO). (b)

Etoposide-mediated induction of cell senescence in MCF-7 cells. Representative images showcasing EdU incorporation in MCF-7 cells at the indicated times after treatment with 20μM etoposide or vehicle (DMSO). Note the strong cell cycle arrest observed in senescent cells (7 days post 20 μM etoposide). (c) We assessed the viability of senescent and non-senescent MCF-7 after treatment with obatoclax for 24 hours. Senescent cells show higher sensitivity to obatoclax compared with non-senescent cells, indicating that obatoclax shows senolytic activity. Cell viability was assed using the alamarBlue metabolic activity indicator. The established senolytic ABT-263 was used as positive control. The mean of three independent experiments (n=3) is shown; error bars indicate standard deviation (SD). (d) Impact of Obatoclax on cell viability in senescent MCF-7. Obatoclax was tested at the indicated concentrations for 24h in cells with or without senescence induction. Cell viability was assessed with Calcein AM green (live cells) and SYTOX blue (dead). Images are representative of 3 independent experiments. Related to Fig. 2.

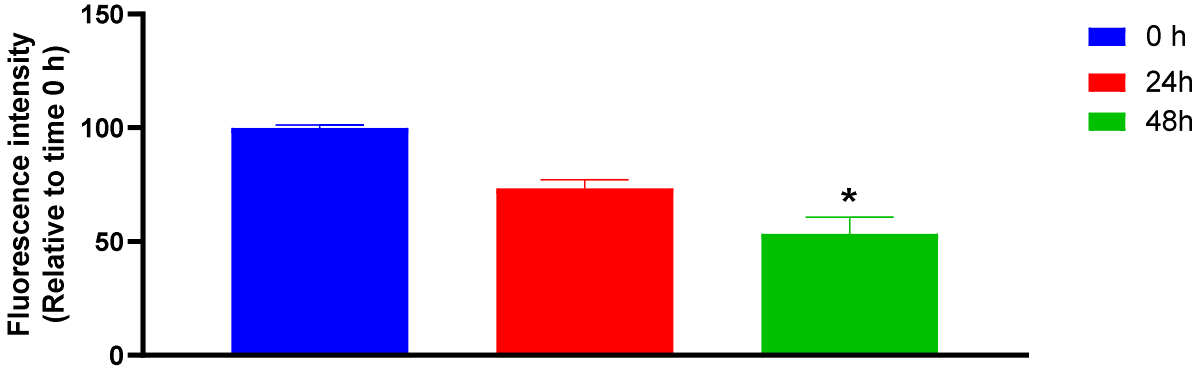

Supplementary Figure S7. Stability of obatoclax. The stability of obatoclax was assessed by measuring the intensity of the compound after incubation at 0, 24, and 48 hours at 37°C. The data was normalized based on the fluorescence intensity at the 0-hour time point. The mean of three independent experiments (n=3) was calculated, and error bars indicate the standard error of the mean (SEM). Treatments were analyzed and compared with those treated at the 0-hour time point using a one-way ANOVA with a Dunnett post-test. * indicates $p < 0.5$. Related to Fig. 4.

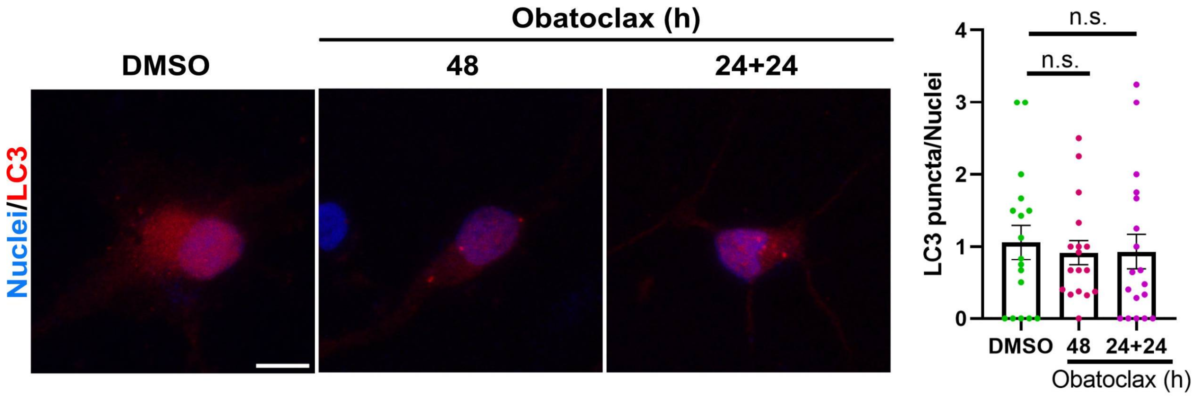

Supplementary Figure S8. Obatoclax induces autophagic efflux in P525L FUS-eGFP iPSC-derived neurons. Confocal fluorescent micrographs showing LC3 puncta in P525L FUS-eGFP iPSC-derived neurons treated with DMSO and obatoclax at 10 nM for 48 hours. The treatment mentioned as 48 hours was treated once with the compound meanwhile the treatment mentioned as 24+24, it was treated twice, each 24 hours, with the compound. The mean of three independent experiments (n=3), error bars indicate SEM. Treatments were analyzed via one-way ANOVA with a Dunnet post-test. n.s. indicates not significant. Scale bar = 10 µm. Related to Fig. 4.

Supplementary Experimental procedures

Chemicals

Product	Supplier	Catalogue number
**Ammonium persulfate (APS)**	Acros organics	401161000
**Bis-Acrylamid mix 30%**	Roth	3029.2
**β-mercaptoethanol (β-ME)**	Sigma-Aldrich	M7522
**Dimethyl sulfoxide (DMSO)**	Sigma-Aldrich	D2650-100ML
**Ethanol**	VWR	20821.321
**Ethidium bromide (EtBr)**	Roth	2218.1
**Formaldehyde 37%**	Roth	CP10.2
**Glycerol**	Sigma-Aldrich	G9012
**Glycine**	Roth	0079.3
**Hoechst 33342**	Thermo Scientific	H3570
**Hydrochloric acid (HCl)**	VWR	20252.244
**Isopropanol (2-propanol)**	Applichem	A3465,2500
**Methanol**	Roth	8388.6
**Milk powder**	Roth	T145.2
**Paraformaldehyde (PFA)**	Science services	E15714-S
**Phosphate buffered saline (PBS)**	Gibco, Thermo Fischer Scientific	14190144
**Potassium Chloride (KCl)**	Sigma-Aldrich	P1597-500g
**Protease inhibitor cocktail**	Roth	4693132001
**Sodium arsenite**	Sigma-Aldrich	1062771000
**Sodium dodecyl sulphate (SDS)**	Roth	CN30.2
**Sodium Chloride (NaCl)**	Roth	3957.3
**Tetramethelthylenediamine (TEMED)**	Serva	35925.01
**Tetradotoxin**	Abcam	Ab120054
**Tris**	Roth	4855.1
**Tris-HCl**	Roth	9090.2
**Triton X-100**	Roth	3051.3
**Tween-20**	Applichem	A4974.0100

Cell culture

Product	Supplier	Catalogue number
**Accutase**	Sigma-Aldrich	AT 104
**B27 plus supplement without vitamin A (100x)**	Gibco	A3582801
**Bovines Serum Albumin (BSA) 7.5 %**	Invitrogen	15260037
**Calcein-AM Red**	Cayman Chemicals	20632
**Dispase**	StemCell Technologies	07923
**DMEM F12**	Gibco, Thermo Fischer Scientific	11320033
**DMEM high glucose**	Gibco, Thermo Fischer Scientific	11965084
**Laminin-521**	Biolamina	LN521-03
**L-glutamine**	Merck Millipore	
**FBS (Fetal Bovine Serum)**	Sigma-Aldrich	C8056
**KnockOut DMEM**	Gibco, Thermo Fischer Scientific	10829018
**Matrigel human Embryonic Stem Cell qualified**	Corning Life Sciences	354277
**Matrigel Growth Factor Reduced**	Corning Life Sciences	354230
**mTESR1**	StemCell Technologies	85850
**N2 supplement (200x)**	Gibco, Thermo Fischer Scientific	17502048
**Neurobasal medium**	Gibco, Thermo Fischer Scientific	21103049
**Non-essential amino acids (NEAA)**	Millipore	K0293
**Opti-MEM medium**	Gibco, Thermo Fischer Scientific	11058021
**Penicillin/Streptomycin/L-glutamine (PSG)**	Biochrom	A2213
**Poly-D-Lysine**	Gibco, Thermo Fischer Scientific	A38904-01
**Poly-L-ornithine**	Sigma-Aldrich	P4957
**ReleSR**	StemCell Technologies	05872
**RIPA Buffer Lysis**	Santa Cruz Biotechnology	SC-24948A
**Serum Replacement**	Invitrogen	10828010

Small molecules

Product	Supplier	Catalogue number
**Ascorbic acid (AA)**	Sigma-Aldrich	A4544
**Activin A**	eBioscience	34-8993-85
**CHIR 99021**	Axon Medchem	Axon 1386
**DAPT**	Cayman Chemicals	13197
**Compound E**	Peprotech	2091746
**dbcAMP**	Sigma-Aldrich	D0627
**DMH-1**	Tocris/Bio-Techne	4126-10
**Dorsomorphin dyhidrocloride**	Selleckem	S7306
**Human Brain derived neurotrophic factor (BDNF)**	Peprotech	450-02-100
**Human Glia-derived neurotrophic factor (GDNF)**	Peprotech	450-10-100
**Murine Fibroblast growth factor (FGF)**	Peprotech	450-33
**Purmophamine (PMA)**	Cayman Chemicals	1009634
**Retinoic Acid (RA)**	Sigma-Aldrich	R2625
**ROCK inhibitor Y-27632**	Selleckchem/Absource	S1049
**SB341542**	Cayman Chemicals	Cay13031-10
**Smoothened agonist (SAG)**	Cayman Chemicals	11914
**Transforming growth factor β3 (TGFβ3)**	Peprotech	AF-100-36E
**Valproic acid (VPA)**	Cayman Chemicals	13033

Kits

Name	Supplier	Catalog Number
**Duolink® In Situ Red Starter kit**	Sigma-Aldrich	DUO92008
**ECL Prime Western Blotting Detection Reagent**	GE Healthcare	RPN2236
**Jess/Wess Separation 12-230 kDa**	Protein Simple	SM-W004-1
**Pierce BCA Protein Assay Kit**	Thermo Fisher Scientific	23225

Antibodies

Product	Host	Dilution	Supplier	Catalogue number
**Bcl-2**	Mouse	1:1000 (WB)	Cell Signaling Technology	15071
**Bcl-2**	Rabbit	1:200 (PLA)	Proteintech	12789-1-AP
**Beclin 1**	Mouse	1:200 (PLA)	Proteintech	66665-1-Ig
**CC3**	Rabbit	1:500 (IF)	Cell Signaling	9661S
**FUS**	Mouse	1:500 (IF)	Sigma-Aldrich	AMAB90549
**GAPDH**	Rabbit	1:4000 (WB)<br>1:2000 (Wes)	Cell Signaling Technology	21185S
**G3BP1**	Rabbit	1:4000 (IF)	Sigma-Aldrich	PA5-29455
**LAMP1**	Rabbit	1:2000(WB)	Cell Signaling Technology	9091
**LC3**	Rabbit	1:1000 (WB),<br>1:200 (IF)	Novus biological	NB600-1384
**p62**	Mouse	1:50 (Wes)	Abcam	ab56416

Secondary antibodies

Product	Dilution	Supplier	Catalogue number
**AlexaFluor 568 Donkey anti-Mouse**	1:1000 (IF)	Thermo Scientific	A10037
**AlexaFluor 647 Donkey anti-Mouse**	1:1000 (IF)	Thermo Scientific	A31571
**AlexaFluor 568 Donkey anti-Goat**	1:1000 (IF)	Thermo Scientific	A11057
**AlexaFluor 647 Donkey anti-Goat**	1:1000 (IF)	Thermo Scientific	A21447
**AlexaFluor 568 Donkey anti-Rabbit**	1:1000 (IF)	Thermo Scientific	A10042
**AlexaFluor 647 Donkey anti-Rabbit**	1:1000 (IF)	Thermo Scientific	A31573
**HRP-coupled anti-rabbit**	1:10000 (WB)	Jackson ImmunoResearch	711-035-152
**HRP-coupled anti-mouse**	1:10000 (WB)	Jackson ImmunoResearch	715-035-150

Proteomics reagents

Instrumentation: Q-EXACTIVE HF - DIA		
Instrument / Parameter	Value	Comments
**Q-Exactive HF**	ThermoScientific, Bremen, Germany	DIA -Mode (positive ion mode)
**MS1**		
Polarity	Positive	
Resolution	R120000 at m/z 200	
AGC	3x 10E6	
Max. Fill Time	50ms	
Lock Mass	m/z 445.120025	Dodecamethylcyclohexasiloxane (Schlosser and Volkmer-Engert, 2003)
Scan Range	m/z 395-900	
Spray Needle	10µm	Fossiliontech,Madrid, Spain
Voltage	2.3-2.7kV	(might vary between experiments)
Spectrum Data Type	Profile	
**MS2-DIA**	**DIA**	**HCD**
Resolution	R15000 at m/z 200	
AGC	1E6	
Max. Fill Time	50ms	
Loop Count	42	
MSX Count	1	
MSX isochronous ITs	On	
Isolation	14.0 m/z	
Isolation window Offset	0.0 m/z	
Scan Range	200-auto m/z	
Fixed 1st Mass	200 m/z-	
Norm. Collision Energy	27	
Spectrum Data Type	Centroid	
**Instrumentation: THERMO DIONEX3000 RSLC**		
Instrument / Material	Manufacturer (Supplier)	Comments

Dionex3000 RSLC	ThermoScientific, Idstein, Germany	Nanoflow System
Acclaim PepMap 100 C18, 3 µm, 300 µm x 5 mm, Acclaim PepMap C18 3, µm, 75 µm x 15 cm	ThermoScientific, Idstein, Germany	Trap-Column Setup<br>Load: 2µl/min<br>Separation: 300nl/min
**Software version**		
Instrument / Material	Manufacturer (Supplier)	Comments
DIA-NN V1.8	V. Demichev	(Demichev et al., 2020)
**SDS gel electrophoresis**		
Item (Chemicals)	Manufacturer (Supplier)	Order Number
Methanol	Merck	1.06018.2500
Coomassie brilliant blue r-250	AppliChem	A1092
Acetic acid 100% p.a.	Carl Roth	3738.1
Water	Merck	1.15333.2500
Tris-Glycine buffer (10x)	Serva	42529.01
Simply Blue Safe Stain	Invitrogen	LC6060
NuPage MOPS SDS Running Buffer (20x)	Novex by life technologies	NP0001
Non reducing lane marker (5x)	Thermo Scientific	39001
2-Mercaptoethanol 99%	Sigma-Aldrich	M3148
PageRuler Plus Prestained Protein Ladder	Thermo Scientific	26619
**In gel digestion**		
Item (Chemicals)	Item (Chemicals)	Order number
DTT	Sigma-Aldrich	D-5545
NH_4HCO_3	Sigma-Aldrich	09830
IAA	Sigma-Aldrich	I-1149
Water HPLC grade	Merck	1.15333.2500
Acetonitrile HPLC Grade	Merck	1.00029.2500 or 1.00030.2500
Formic Acid p.a.	Merck	1.00264.0100
Trypsin Gold sequencing grade, (modified Trypsin)	Promega	V5280

Electrophysiology

Standard MEA chambers (60MEA200/30iR-TI-gr, Multichannel Systems) were coated with Poly-*D*-lysine (Gibco) and incubated overnight at 37°C. The chambers were washed three times with sterile ddH_2O and dried. After the chambers were coated with Laminin (LN521, Biolamina) and incubated overnight at 4°C. Neurons were seeded (400, 000 cells per MEA chamber) after the patterning and Activin A induction (day 8). The cells were cultured with maturation medium (N2B27, Ascorbic acid, TGF-β, dibutyryl cyclic-AMP sodium salt, BDNF and GDNF). Media was change every third day. Electrophysiological measurements took place on DIV21 and DIV23. On DIV21 only baseline recordings were obtained. On DIV23 pharmacological manipulations took place. To assess the involvement of voltage-gated sodium channels in spiking activity, action potential firing was assessed in the presence of tetrodotoxin (TTX, 1 µM, Abcam) and following wash out of TTX. All media changes took place in a safety cabinet (Herasafe KS12; Thermo Electron LED GmbH, Langenselbold, Germany) and addition of new medium was always performed slowly to not disrupt the electrode-cell connection. All incubations took place in the aforementioned incubator where cells were maintained leading up to and between recordings.

To introduce TTX after completion of the baseline recording, all medium was removed and pre-warmed medium, with freshly added TTX (stock concentration 1 mM), was immediately introduced to the recording chamber. Following an incubation of 10 min at 37°C, the TTX recording was commenced within 15 min following the addition of TTX. Subsequent to termination of the recording, medium containing TTX was removed, which was realized within 30 min following its introduction. To ensure the removal of TTX, the freshly added medium, was removed immediately and replaced by new medium yet again. With this, the chip was returned to the incubator for 10 min. The wash out recording was then commenced within 15 min following the addition of fresh medium.

Cells growing on the aforementioned chips were visually inspected with an inverted microscope (Eclipse Ti-E, Nikon Europe B.V., Amstelveen, The Netherlands). Images were taken at 2x to 30x magnification using an interline CCD camera (Clara, Andor, Belfast, Northern Ireland). These images were used to assess whether live cells populated the area within a 20 µm radius surrounding the 40 µm diameter electrode-bed, i.e. within 30 µm surrounding the electrode. Electrophysiological signals were obtained for 10 minutes at 37°C, amplified 1100x and sampled at 25 kHz using a 60 channel microelectrode array recording system (MEA1060-Inv-BC, MCS) mounted on the aforementioned microscope.

Electrophysiological recordings were analyzed offline (Offline Sorter, Plexon Inc, Dallas, Texas, USA). The signal was high pass filtered at 300 Hz (4-pole Butterworth) and event were detected using -5.0 standard deviations (SD) as threshold. Extracted waveforms had a duration of 3.6 ms, including a pre-threshold period of 1.4 ms and a dead time of 2.2 ms. Sorting was performed using valley seeking (Parzen Multiplier set to

one and with removal of outliers exceeding 2 SD) and peak alignment was applied. Distinct waveforms from individual electrodes were not assessed differentially but treated as multiunit. Measures of variations are SD.

Senolytic activity evaluation

MCF-7 cells, kind gift from Anna Taubenberger's lab, were induced into senescence by exposure to 20µM etoposide for 7 days. Proliferating controls were treated with DMSO. Senescence-associated beta galactosidase (SAbgal) and EdU stainings were performed as previously described (Walters et al.). Obatoclax treatment was performed at the indicated concentrations for 24h on control (DMSO treated) or etoposide-induced senescent cells seeded at similar confluence. Viability was assessed through a metabolic activity indicator (alamarBlue) or vital dye incorporation (Calcein green/Sytox Blue) as per manufacturer's instructions. Detection of alamarBlue was performed on a GloMax Discover plate reader. Imaging was performed on a Nikon fluorescence microscope.

Obatoclax staibility

Obatoclax were diluted at 100 µM in water and incubated at 37°C for 0, 24 and 48 hours. The blank (water) and the obatoclax solution was measured using a 490 nm excitation filter and a 600 nm emission filter on a Biotek Synergy™ NEO microplate reader.

References

Demichev, V., Messner, C.B., Vernardis, S.I., Lilley, K.S., and Ralser, M. (2020). DIA-NN: neural networks and interference correction enable deep proteome coverage in high throughput. Nat Methods *17*, 41-44. 10.1038/s41592-019-0638-x.

Schlosser, A., and Volkmer-Engert, R. (2003). Volatile polydimethylcyclosiloxanes in the ambient laboratory air identified as source of extreme background signals in nanoelectrospray mass spectrometry. J Mass Spectrom *38*, 523-525. 10.1002/jms.465.

Walters, H.E., Troyanovskiy, K.E., Graf, A.M., and Yun, M.H. Senescent cells enhance newt limb regeneration by promoting muscle dedifferentiation. Aging Cell *n/a*, e13826. https://doi.org/10.1111/acel.13826.
